# ‘Harnessing the power of the law’: a qualitative analysis of the legal determinants of health in English urban planning and recommendations for fairer and healthier decision-making

**DOI:** 10.1186/s12889-023-15166-0

**Published:** 2023-02-11

**Authors:** Lisa Montel

**Affiliations:** Centre for Health, Law and Society, University of Bristol Law School, 8-10 Berkeley Square, BS8 1HH Bristol, UK

**Keywords:** Legal determinants of health, Structural determinants, Upstream determinants, Urban development, Urban health, Local government

## Abstract

**Background:**

Urban environments impact negatively on the risks of non-communicable diseases and perpetuate health inequalities. Against this, law could play a critical role, notably through implementing and securing visions of health and well-being, and evidence-based interventions.

**Methods:**

Seven teams conducted 123 interviews with 132 actors in urban planning in England. Teams had expertise in urban planning, transport, real estate, public health, public policy, administration, and management. An additional team with expertise in law analysed data from all interviews to explore how the law is perceived and used to promote health in urban planning.

**Results:**

Six issues were identified as preventing actors from using the law to improve health in urban planning: (i) density and complexity of the law; (ii) weak and outdated regulatory standards; (iii) absence of health from legal requirements in the decision-making process; (iv) inconsistent interpretations by actors with competing interests; (v) lack of strong health evidence-based local planning policies; and (vi) inertia of the law.

**Conclusions:**

The legal determinants of health listed in the *Lancet*-O’Neill Commission’s report need to be strengthened at the local level to effectively deploy law in English urban development. The findings call for strong, evidence-based local planning policies and decision-making frameworks, placing health as (one of the) core value(s) of urban planning and showing what types of development benefit health, i.e., prevent NCDs risks and reduce health inequalities on the long term. The legal capacity of local government should be strengthened to empower decision-makers in shaping urban development that promotes health for everyone.

## Background

In 2018, 55% of the world population (4.2 billion) lived in urban areas [1]. This proportion is expected to increase to more than 68% by 2050 [1]. Urban environments impact on the risks of ill health, notably non-communicable diseases (NCDs). This is because urban planning organises critical determinants such as access to green space, road traffic, cycle lanes, access to healthy and affordable food, as well as proximity of alcohol and tobacco sales. These determinants impact on physical activity, outdoor air quality, diets, tobacco and alcohol consumption, which are all factors of the four major NCDs, i.e. cancer, diabetes, respiratory diseases and cardio-vascular diseases [2–5]. Recent literature reviews suggest that both risks and incidence of mental illnesses are higher in cities than in rural areas [6, 7]. Beyond impacting NCD risks and mental health, urban environments also create and perpetuate health inequalities through the social determinants of health [8–10]. Deprived areas, often segregated from affluent areas, present more air pollution and poorer health outcomes [11]. Urban environments have moved up the global development agenda with Sustainable Development Goal (SDG) 11 on inclusive, safe, resilient and sustainable cities [12, 13]. SDGs all impact one another, including SDG 11 with SDG 3 on ensuring healthy life and well-being, SDG 6 on clean water and sanitation, and SDG 10 on reduced inequalities [14]. In other words, promoting health and well-being in urban environments is not only important for the impact on health alone, but also structurally to remedy health and social inequalities.

Urban planning in England operates within several legal and regulatory environments, each requiring its own expertise. At the forefront, urban planning is governed by planning law statutes such as the Town and Country Planning Act 1990 and the Planning Act 2008. Planning law itself must be read in conjunction with other regulatory environments, such as the Building Act 1984 and the Regulations from that Act. Running in parallel, there are legal instruments that do not strictly belong to planning law but that govern decisions within urban planning, such as environmental regulations imposing standards of clean air and retrofitting of buildings. In addition, actions of private and public actors are regulated by the law of contract, corporate law and, when relevant, procurement law. Actions and decisions of public actors are themselves governed by public law, including statutes such as the Local Government Act 2010, the Equality Act 2010 and the Health and Social Care Act 2012. This broad picture is far from being complete, but it provides an overview of the density and complexity of the legal and regulatory environments governing urban planning in England. Within this, Local Planning Authorities (LPAs) make local development plans. These plans must respect the National Planning Policy Framework (NPPF) – the national policy in England guiding all urban planning decisions [15]. In turn, those local development plans are used as reference points both by developers who wish to put forward new planning applications and by officers in LPAs to make decisions on these applications. All new projects must respect the local development plan in force in a given area or, when such plan does not exist, the NPPF (NPPF § 11(c)-(d) &47).

Within urban environments themselves, the law plays a critical role. Burris and Lin identified three ways law can be used by local authorities to improve health: the law may be used as a tool to change individual behaviour, through for example creating non-smoking zones or measures to reduce air and water pollution; as a target of health promotion efforts, such as changing restrictive abortion laws or punitive drug laws that have negative consequences on health; and to (re)organise governance, including by taking a health-in-all policies approach and fostering collaboration between departments such as public health and safety – or for this study, planning [16]. Using the law to reduce health inequalities and promote well-being is anchored in literature on the legal determinants of health [17]. The *Lancet*-O’Neill Commission’s report on legal determinants shows that the ‘power of the law’ may be ‘harnessed’ – in the words of the Commission – to improve health outcomes and remedy health inequalities in at least four domains: (i) by translating (health policy) vision into action; (ii) by strengthening governance; (iii) by implementing fair, evidence-based health interventions; and (iv) by building legal capacity for health [17]. Law is also an upstream determinant of the urban planning system. As such it impacts health, including the creation and perpetuation of health inequalities. However, despite a well-documented role of the law to reduce health inequalities and promote well-being, studies have called for more research on the impact at the local level [16, 18]. How is the law ‘activated’ at the local level in English urban planning? How is it perceived and used (if at all) by actors in urban development to promote health? This study is necessary to supplement research on the legal determinants of health with empirical data and understand how to deploy law to improve urban health at the local level [16, 17].

## Methods

Between May and October 2021, 123 interviews were conducted with 132 key stakeholders as part of the transdisciplinary project [name redacted for review] [19, 20].

Interviews were conducted by seven disciplinary teams with expertise in public health, urban planning, real estate, transport, public policy, management and public administration. A team with expertise in law designed a ‘law question’ to be asked to all interviewees (Table 1). The aim of which was to explore how the law is perceived and used to promote health (or not) in English urban planning decision-making. Participant responses to this question were used to generate the findings for this paper.

**Table 1:** The ‘law question’ asked to 132 interviewees

• ‘How (if at all) do legal considerations influence decision-making?’
Prompts:
• ‘Are you worried about the possibility of legal challenges?’
• ‘Do you limit your compliance with the law to the minimum standards, or do you go above and beyond?’

Participants were identified through purposive sampling. First, a database of approximately 500 actors was created, where potential participants were evaluated against two selection criteria (i) the individual’s understanding of the urban development system; and/or (ii) their influence in the system. These actors were identified through desk-based searches, literature reviews, stakeholder mapping and a pilot project [21]. Second, each team reduced their initial invitee list to a maximum of ten individuals who best met the criteria, and third engaged in snowball sampling to maximise system coverage [22]. Ultimately, the participants interviewed covered a wide range of roles in urban planning, such as local government officers, local government elected members, national government actors, real estate actors, property developers and consultants.

All law-related data were saved by each of the seven interview teams under a ‘law category’ in NVivo 12 [23]. Guidance was provided so that data would be organised consistently under the law category between each disciplinary team. The data under the law category was then analysed by the author using reflexive thematic analysis [24, 25]. First, deductive codes were developed by the law team to organise the data into areas that were identified as focus points in the previous phase of the project, e.g. ‘risks of legal challenges’, ‘accountability’ or ‘engagement with and interpretation of the law’ (Table 2).

**Table 2:** List of deductive codes

• Accountability
• Areas of legislation
• Changes to planning law
• Engagement with and interpretation of the law
• Landmark cases
• Legal barriers to healthy urban development
• Risks of legal challenges
• Legal duties and targets in decision-making
• Legal tools to promote health

The data in each category was further coded inductively through an iterative process. Examples of these inductive codes include ‘perceived threat of a legal challenge’ and ‘reasons or motivations to bring a legal challenge.’ Recognising that ‘the researcher’s role in knowledge production is at the heart of [the] approach’ in reflexive thematic analysis, the law researcher developed themes from the data [24]. Themes are understood as ‘interpretive *stories* about the data, produced at the intersection of the researcher’s theoretical assumptions, their analytic resources and skill, and the data themselves’ [24]. Six of the themes developed from the data are presented in the Result section below and discussed in this paper. Burris and Lin’s broad understanding of the law was used to analyse the data, i.e. law encompasses ‘legal texts like constitutions and statutes, but also the formal policies of public and private institutions, the implementation/enforcement practices of legal agents and the beliefs about the law prevailing among those subject to it’ [16]. The method of analysis and findings from all disciplines are discussed in two separate papers [26, 27]. All methods were carried out in accordance with relevant guidelines.

## Results

Six themes emerged from the data, suggesting six barriers that limit the use of the law to promote health in English urban planning decision-making, as perceived by the interviewees. The quotes below belong to 16 different stakeholders, with expertise in property development [2], urban planning [5], finance [4], transport [1], public health [3] and politics [1]. Eight interviewees come from the private sector and eight from the public sector, including six at local or regional level and two at the national level.

1. Density and complexity of the law.

The interviews show that key actors such as planning officers in LPAs, developers, consultants and real estate investors see the law as dense, complex and difficult to keep up to date with. The quotes below illustrate how actors find the law complex across several planning domains, such as determining the financial viability of new projects or evaluating the degree of freedom allowed when designing new projects within a highly regulated environment.

*“[I]t is remarkably hard to say anything here about us having a proactive planning system. Everything is considered to be a burden on the market – it’s a regulatory burden.” (TC-242, senior policy officer, housing)*

*“The challenge I guess is that the law is really complicated and really dense and all those sorts of things. So, we do lots of training around law, civil engineering law and contract procedures (…). Understanding law and changes in law is sometimes a challenge and I think a lot of our training around work is around keeping up to date with law (…). So, it’s really important, it’s quite hard to keep up and keep going with it” (TM-113, consultant, urban planning/transport)*

*“[T]he rules on viability […] are horrifically technical, very obscure, most people don’t understand them at all, but have a huge determining effect on the pattern of development and what gets built. And then, how they’re interpreted by case law – individual cases will have a massive impact.” (AS-192, consultant, housing and finance)*

2) Weak and outdated regulatory standards.

In addition to the complexity of the legal environments surrounding urban planning, interviewees felt that some regulatory standards are not enough or not adapted to implementing critical goals to move towards healthier urban areas. For instance, the net-zero strategy [28] and building regulations are deemed insufficient to achieve safety and well-being of residents.

*“I guess that’s things like net zero emissions by 2050, 2040, whatever are great but [sighs] you know, there needs to be a bit more than that.” (AS-180, investor, urban planning)*

*“Grenfell is the result of twenty years of failing of building regs. We all knew that they weren’t good enough, but you’ve created a culture of ‘build it to regs. That’s good enough.’ And obviously it’s not. That’s the minimum standard.” (JB-199, property developer)*

3) Absence of health from legal requirements in the decision-making process.

In contrast with the heavily regulated environments that urban planning operates in, critical values of health and well-being, less still health inequalities, are not integrated into the legal requirements that LPAs rely on to base their decisions. If such values are mentioned, it is sporadically, and they are ill-defined.

*“So, if for the future we are starting to say, ‘well look, sustainable development is so much more and real health outcomes are a key material consideration*^1^*in determining planning applications’, I would love that, but my sense is at the moment we haven’t got the backing for that, we haven’t got the national guidance or the national legislation and also local plan and policies for that.” (WB-331, local government, development management)*

The absence of health in urban planning decision-making processes was felt even when the broader field of public law was considered.^2^ Some interviewees mentioned the statutory duty of local authorities to improve the health of people in their jurisdiction,^3^ but indicated that it was neither considered in planning decision-making, nor was it used as a basis to challenge decisions on appeal or in judicial review.

*“There is a duty on local authorities to promote and protect the health of the population. So taking health impacts into account in the short, medium and longer-term (…) local authorities could be challenged. (…) the statutory duty of the authority is to be assured that the health of the public is protected and promoted. So that could be used as a lever.” (WA-292, local government, public health)*

A similar argument was made with the equality duty under the Equality Act 2010 [29].

*“[W]e are fundamentally failing our equality duty almost everywhere on the network because we still have barriers to access transport. So, that’s walking, cycling, public transport. I’m astounded that that hasn’t been challenged more. I think that it will be eventually, because the legislation is in place, it’s been in place for long enough that reasonable adjustments should have been made and aren’t, and they continue not to be (…).” (BX-596, urban/transport planner)*

4) Inconsistent interpretations by actors with competing interests.

The above remarks about the legal and regulatory environments operating within, and in parallel to, urban planning lead to inconsistent interpretations of the law by different actors, each trying to uphold differing interests. Commercial actors, notably developers, are in principle profit-oriented, which means that financial returns guide their decision.

*“[I]f you look at Grenfell, which is a very rare example (…) [developers] don’t just try and comply with the regulations. They try and game and avoid and break the regulations. So it doesn’t matter whether you’re talking planning or building regulations. A significant part of the built environment industry is trying to find ways to minimise the impact of those regulations on their profits.” (JM-184, property developer/investor)*

Values relating to health promotion, including those within the Biodiversity Net Gain approach to development,^4^ are considered a financial risk by the industry.

*“It’s being seen as a risk I think generally. That obligation is seen as onerous by the industry probably, however, I think the industry has sort of got used to the biodiversity (…). It’s been on the agenda for long enough. I think the good thing is though, again if it’s thought about early enough, then actually you can create really pleasant places if they have the sorts of spaces within them that will deliver that agenda. I like the long-term bit because I think that ensures that those things don’t just become denuded over time (…).” (Property developer, RSL, GN-190)*

*“[I]f they [developers] know about [the Biodiversity Net Gain] at the time of making an offer or considering buying the site, they can factor that in to the price and the bid, and therefore it will be embraced and welcomed rather than resisted for viability reasons.” (Planning consultant, RB-179)*

When it comes to the planning inspectorate – the body reviewing appeals against a refusal to grant planning permissions – health considerations seem to be interpreted inconsistently. Two interviewees from local councils explained how the lack of certainty on how health or the public health duty would be considered at the planning inspectorate discouraged LPAs from refusing planning permissions on health grounds.

*“[There is] a question in the minds of planning officers as to whether a planning inspector would uphold a rejection of a complaint on health grounds. So they might well feel that they could understand the objection, they might even sympathise with the objection, but they didn’t feel they could necessarily advise the committee to endorse the objection when they were not sure if the planning inspector would uphold that. They felt the planning inspectors would go down more conventional approaches rather than take account of the health issue. That declined as a problem when health became included in the national planning policy framework but it didn’t go away because there was still a feeling that planning inspectors hadn’t necessarily fully clocked the significance of health and even though it was in the NPPF they didn’t necessarily see it as the same overriding issue (…).” (XE-990, former director of public health)*

*“I think that the challenge is that (…) sometimes (…) planning inspectors will say that kind of action [legal challenges on the basis of the public health duty] is best done through non-planning systems rather than the planning system and therefore (…) you get planning inspectors saying different things and (…) interpret law in different ways.” (DB-243, urban/transport planner)*

5) Lack of strong health evidence-based local planning policies.

Interviewees reported the critical importance of planning policies based on (local) evidence of urban health benefits, because such policies would be used to justify a decision if it were to be brought before the Planning Inspectorate.

*“I think from a planner’s perspective, obviously it’s the cost of appeals that is the concern (…). [I]f we don’t have robust policies in place informed by robust evidence then you’re more likely to get an appeal happening.” (UT-507, sustainability senior officer)*

*“What matters is whether or not someone points out, something pertinent in relation to our policy [when submitting a planning application]. That’s all that matters because actually there is always hanging over us, that the applicant has a right to take this on to the inspector. We can’t make decisions that wouldn’t stand up when it goes to the inspector. That is a matter of policy and the application before us.” (BL-533, elected official, urban planning)*

6) Inertia of the law.

Unclear or outdated policies, as well as the absence of health promotion in urban planning policies, are difficult to change because local governance systems do not allow for flexibility.

*“Just the acceptance of a recommendation by an organisation, whether it be a health body or a council, it’s very hard for that to break through into professional practice, especially when there are codes of policy which would have to be changed (…). For example, planners would say to me well you may have got this resolution from the council but the fact is the development policies still say this and the process of changing them would consist of drafting a change, advertising a change, hearing objections, there could be an appeal from a planning inspector, so the decision of the council can’t change any of that unless we go through that process. So I think planning is particularly trapped by the fact that it operates in a legalistic framework in which many of these things which we see as part of the public health process are not part of that formal legal process.” (XE-990, former director of public health)*

## Discussion

The *Lancet*-O’Neill Commission’s report on legal determinants shows that the law may be deployed to improve health outcomes and remedy health inequalities in at least four domains: (i) by translating (health policy) vision into action; (ii) by strengthening governance; (iii) by implementing fair, evidence-based health interventions; and (iv) by building legal capacity for health. The six barriers suggested by the interviewees show that the power of the law could be better harnessed to promote health in English urban planning.

Several interviewees deplored the lack of clear policy and the absence of health from decision-making processes to guide their decisions in a way that would promote well-being. Translating vision into action means that urban planning policies should integrate health as a core value and give guidance to weigh health matters against other considerations, such as financial viability of development projects.

The legal principle to base planning decisions on is the presumption of sustainable development (NPPF § 11). This presumption applies at two levels. At the plan-making stage, LPAs must promote sustainable development, taking into account and addressing local characteristics such as housing needs and effects of climate change (NPPF § 11(a)&(b)). At the decision-making stage, LPAs must approve planning applications if they comply with up-to-date local planning policies (NPPF § 11(c)). Therefore, the quality of development depends on the quality of local development policies.

The NPPF includes values of health and well-being, but our findings suggest that it is unclear how these values should be implemented at the local level. The ‘social objective’ of urban planning in England (NPPF § 8(b)) includes ‘vibrant and healthy communities’, but these communities are defined as the number of homes and as ‘well-designed, beautiful and safe places, with accessible services and open spaces’ (NPPF § 8(b)). Further in the NPPF, health and well-being are integrated, yet interviewees did not feel that the NPPF – which applies nationally – constitutes a strong policy basis to promote health in their *local* area. For instance, urban development should promote social interactions, safe streets, and access to infrastructure encouraging physical activity and healthy food (NPPF § 92(c)). Building standards and air pollution must also be considered (NPPF § 185). These findings must be tempered by the revision of the NPPF in July 2021, which improved provisions on sustainable transport, active travels, as well as well-designed places [30].

The reading of the NPPF and of the interview data show that the problem lies at two levels. First, health and well-being values are not sufficiently integrated into planning law (including national and local planning policies). Then, when such values are, there is not enough guidance for decision-makers to deploy health at the local level. Questions such as ‘what does healthy development look like in a given area’ and ‘how to implement it’ are not answered through planning policies.

A few legislative changes in the UK seek to fill this gap. The Biodiversity Net Gain was included as a requirement for planning in the Environment Act (2021) [31]. This was seen by one interviewee as an improvement in planning, whilst considered a financial risk by one developer. Remarks from the latter interviewee show how eventually the financial risk associated with Biodiversity Net Gain could be reflected on a development’s value – and therefore on housing prices – perpetuating health inequalities.

The Healthy Homes Bill (HHB), being discussed in the House of Lords at the time of writing this article, also seeks to address the lack of health and well-being values in planning law [32]. If passed as law, the new Act would require planning policies to include the 11 ‘healthy homes principles’ including minimum liveable space; access to natural light; inclusivity, accessibility and adaptability of new homes to the needs of everyone; access to sustainable transport and walkable services; resilience to climate change; freedom from noise and light pollution; and minimisation of the harmful impacts of air pollution (HHB section 3). Responsible authorities, including LPAs, the planning inspectorate and urban development corporations, would need to have due consideration of the 11 principles in the exercise of their duties (HHB sections 5(2) & 5(3)). Through this Bill, elements of the public health duty and the equality duty would be integrated formally into planning law.

Further, interviewees exposed the lack of health-related evidence at the local level to inform planning policies and decisions. This links to another legal determinant from the *Lancet*-O’Neill report to implement fair, evidence-based health interventions. LPAs need evidence showing the health (dis)benefits of policies in urban planning to guide policies and decisions, and to have a robust basis to justify such decisions in the event of a legal challenge. Evidence of the health impacts of planning policies would also reduce conflicting interpretations of planning policies and guide implementation of a common vision of health.

The findings above show that key actors in LPAs lack certainty as to how the planning inspectorate would interpret the reasonableness of their decisions. Following recommendations from the *Lancet*-O’Neill report, officers working in LPAs and the planning inspectorate may be trained and empowered to use the law in a way that consistently promotes health in urban planning. Increased legal capacity for health would also allow local government to improve health at local level in the three ways postulated by Burris and Lin: to change individual behaviour; to target health promotion efforts; and to (re)organise governance [16].

The latter point brings us back to the *Lancet*-O’Neill legal determinant on strengthening governance. Interviewees deplored the inertia of local policies, which cannot be amended easily, discouraging actors in local government to change the rules. Inertia is also noticeable more extremely at the national level, where laws cannot be changed by actors in urban planning. For instance, the mayor of Greater Manchester, its transport commissioner and civil society organisations campaigned in 2021 to amend legislation and simplify the implementation of zebra crossings, which would encourage walking. Currently, such crossings require zigzag markings and flashing lights, and may cost up to £40,000 [33]. These two types of inertia are problematic when legal and regulatory requirements hinder decisions favouring healthy urban development. The barriers discussed in this paper, i.e. policy inertia, lack of resources in LPAs, lack of clear local policy goals integrating health and well-being, lack of local evidence showing the impacts of development on health, and risks of having decisions taken to the Planning Inspectorate may profit some actors. In this context, large property developers and other powerful private actors are left with the advantage of using the law to promote projects that generate the most profits, to the detriment of health and well-bring of residents. These actors may even lobby against further regulations, against clarification of existing regulations, or new standards in favour of reduction of air pollution and promotion of health.

In summary, the legal and regulatory environment surrounding urban development encompasses a plurality of legal domains which renders it complex and dense. Because of this, it is difficult for actors to directly engage with the law. Within that, some regulatory standards, such as standards around clean air and building safety, are seen as too weak or outdated to effectively promote health. Actors in LPAs must base their decisions on the NPPF and local planning policies. However, these instruments do not give enough detail for health to be weighed against other considerations at the local level. When health is mentioned, healthy development is not clearly defined and often limited to provisions of affordable housing. This lack of clarity leads to inconsistent interpretations of the law by different actors with competing interests. Developers, who are profit-oriented, tend to prioritise financial gains whilst LPAs are required to promote the public interest, including public health. The law is also interpreted inconsistently by the planning inspectorate, which discourages LPAs from refusing planning permissions on health grounds. Such tensions call for strong, evidence-based local planning policies and decision-making frameworks, placing health as (one of the) core value(s) of urban planning and showing what types of development benefit health, i.e., prevent NCDs risks and reduce health inequalities on the long term. The legal capacity of local government, in particular LPAs, should be strengthened to empower decision-makers in shaping urban development that promotes health for everyone.

### Recommendations

Following from these challenges associated with the legal determinants of health in urban planning, and informed by the *Lancet*-O’Neill Commission’s proposed actions on the legal determinants of health, four recommendations may be implemented at local and national levels by policy-makers and professional bodies:

1. Incorporate health and well-being considerations in planning law, national and local evidence-based planning policies. One such example are the 11 principles from the Healthy Homes Bill [32]. Such a process should be informed by a transdisciplinary, intersectoral approach involving epidemiologists, urban planners, developers, architects, public health policy-makers, health ethics experts, and professional bodies such as the Royal Town Planning Institute, the Royal Institution of Chartered Surveyors, the Royal Institute of British Architects, and the Institute of Environmental Management and Assessment.
2. Include health and well-being considerations in planning decision-making processes. An example cited by Burris and Lin is the Public Health and Wellbeing Act in Victoria, Australia, that requires local governments to address the social determinants of health through intersectoral planning [16, 34]. One significant addition would be to include the public health duty and equality duty into planning law decision-making and as legal requirements to be considered by the planning inspectorate.
3. Train LPAs, the planning inspectorate, and developers to interpret the (amended) law consistently to promote healthy urban development, including the consistent interpretation of policy. This process would also be informed by a transdisciplinary, intersectoral approach, as in previous points.

### Limitations

The nature of this study as being part of a large transdisciplinary project must be considered. The law question was written by the law team but asked by seven different interview teams without expertise in law. The variety of stakeholders means that the number of interviewees representing each stakeholder category (sector and field) remains small. The aim was to capture how the law is used and perceived by key actors in the urban development system in England, hence why the variety of stakeholders was prioritised over large numbers in fewer categories. Furthermore, data were analysed by a researcher with expertise in law and public health, with their own subjectivity, assumptions and disciplinary biases. This is made explicit in the method and is considered a resource in the reflexive process to interpret the data, as opposed to a weakness [24]. The analysis presented in this paper represents a subjective and disciplinary interpretation of the data.

## Conclusion

This study identified six challenges about the law as perceived or used by key stakeholders in English urban planning. These issues are preventing local governments from deploying the law to implement visions of health equity and well-being in urban environments, which have been identified as critical determinants of health [8, 13, 16]. Law – widely defined as common law, statutes, regulations and policy, systems of governance and encompassing the ‘beliefs (…) among those subject to it’ [16] – is both a tool of power to improve health outcomes, and sometimes a factor of poor health outcomes [17]. By identifying core challenges preventing from ‘harnessing the power of the law’ [17] in English urban planning and providing some recommendations, this article adds to the literature on the legal determinants of health by relying on empirical data.

## Data Availability

The data supporting the findings reported in this paper comprises primary interview data and secondary existing data. A redacted and anonymised version of all primary interview data used in this paper will be made available via the University of Bristol Research Data Repository data.bris two years after the completion of the project, Tackling the Root causes Upstream of Unhealthy Urban Development (TRUUD). All secondary existing data used in this paper is openly available at locations cited in the ‘References’ section of this paper.

## Abbreviations

LPAs: Local Planning Authorities
NCDs: Non-Communicable Diseases
NPPF: National Planning Policy Framework 2012 (as revised in 2021)
SDGs: United Nations Sustainable Development Goals

## Footnotes

1 ‘A material consideration is a matter that should be taken into account in deciding a planning application or on an appeal against a planning decision.’ Planning Portal. Available at: https://www.planningportal.co.uk/services/help/faq/planning/about-the-planning-system/what-are-material-considerations

2 Public law is the body of law that governs public institutions and relations between public bodies and individuals.

3 The statutory duty comes from the National Health Service Act 2006 c.41, Sections 13G and 14T, as inserted by the Health and Social Care Act 2012 c.7.

4 Sects. 98–101 of the Environment Act (2021) contain provisions for the Biodiversity Net Gain in planning. ‘Biodiversity Net Gain is an approach to development, such as a new building or construction, that leaves biodiversity in a better state than before. Where a development has an impact on biodiversity, developers will need to provide an increase in appropriate natural habitat and ecological features over and above that being affected.’ Greater Manchester Combined Authority. Available at: https://www.greatermanchester-ca.gov.uk/what-we-do/environment/natural-environment/biodiversity-net-gain/.

## References

[1] United Nations Department of Economic and Social Affairs. World Urbanization Prospects 2018 2018 [Available from: https://population.un.org/wup/Download/ (Accessed 1 June 2022).

[2] Cavill N, Davis A, Cope A, Corner D. Active Travel and Physical Activity Evidence Review. 2019.

[3] Newby DE, Mannucci PM, Tell GS, Baccarelli AA, Brook RD, Donaldson K (2015). Expert position paper on air pollution and cardiovascular disease. Eur Heart J.

[4] Ortiz C, López-Cuadrado T, Rodríguez-Blázquez C, Simón L, Perez-Vicente R, Merlo J (2022). Physical and social environmental factors related to co-occurrence of unhealthy lifestyle behaviors. Health Place.

[5] Samitz G, Egger M, Zwahlen M (2011). Domains of physical activity and all-cause mortality: systematic review and dose-response meta-analysis of cohort studies. Int J Epidemiol.

[6] Gruebner O, Rapp MA, Adli M, Kluge U, Galea S, Heinz A (2017). Cities and Mental Health. Dtsch Arztebl Int.

[7] Ventriglio A, Torales J, Castaldelli-Maia JM, De Berardis D, Bhugra D (2021). Urbanization and emerging mental health issues. CNS Spectr.

[8] Marmot M, Friel S, Bell R, Houweling TAJ, Taylor S (2008). Closing the gap in a generation: health equity through action on the social determinants of health. The Lancet.

[9] Marmot M, Allen J, Boyce T, Goldblatt P, Miorrison J. Health Equity in England: The Marmot Review 10 Years On. 2020. 10.1136/bmj.m693 32094110

[10] Marmot M, Allen J, Goldblatt P, Herd E, Morrison J. Build Back Fairer: The COVID-19 Marmot Review. 2020.

[11] Brunt H, Barnes J, Jones SJ, Longhurst JWS, Scally G, Hayes E (2016). Air pollution, deprivation and health: understanding relationships to add value to local air quality management policy and practice in Wales, UK. J Public Health.

[12] United Nations. Transforming our world: the 2030 Agenda for Sustainable Development [Internet]. 2015 [cited 2023 Feb 6]. Available from: https://www.unfpa.org/resources/transforming-our-world-2030-agenda-sustainable-development#:~:text=%22We%20resolve%2C%20between%20now%20and,protection%20of%20the%20planet%20and.

[13] Habitat UN, World Health Organisation (2016). Global report on urban health: equitable healthier cities for sustainable development.

[14] United Nations Department of Economic and Social Affairs. The Sustainable Development Goals report: interlinked nature of the Sustainable Development Goals. 2018.

[15] Ministry of Housing, Communities & Local Government.National Planning Policy Framework 2012 (2021 revision) [Internet]. 2021 [cited 2023 Feb 6]. Available from: https://www.gov.uk/government/publications/national-planning-policy-framework--2.

[16] Burris S, Lin V (2021). Law and urban governance for health in times of rapid change. Health Promot Int.

[17] Gostin LO, Monahan JT, Kaldor J, DeBartolo M, Friedman EA, Gottschalk K (2019). The legal determinants of health: harnessing the power of law for global health and sustainable development. The Lancet.

[18] Ibrahim JK, Sorensen AA, Grunwald H, Burris S (2017). Supporting a culture of evidence-based policy: Federal Funding for Public Health Law evaluation research, 1985–2014. J public health Manage practice: JPHMP.

[19] Black D, Ayres S, Bondy K, Brierley R, Campbell R, Carhart N, et al. Tackling Root Causes Upstream of Unhealthy Urban Development (TRUUD): Protocol of a five-year prevention research consortium. Wellcome Open Research. 2021;6(30). 10.12688/wellcomeopenres.16382.1 PMC9296993 35919506

[20] Tackling the Root Causes Upstream of Unhealthy Urban Development (TRUUD). Available from: https://truud.ac.uk/. Accessed 25 Aug 2022.

[21] Black D, Pilkington P, Williams B, Ige J, Prestwood E, Hunt A, et al. Overcoming Systemic Barriers Preventing Healthy Urban Development in the UK: Main Findings from Interviewing Senior Decision-Makers During a 3-Year Planetary Health Pilot. Journal of urban health: Bulletin of the New York Academy of Medicine. 2021;98(3):415-27. 10.1007/s11524-021-00537-y PMC8190222 33939069

[22] Bazeley P. Qualitative data analysis: practical strategies. 1st ed. London: SAGE Publications Ltd; 2013.

[23] QSR International Pty Ltd. NVivo (version 12). 2018.

[24] Braun V, Clarke V (2019). Reflecting on reflexive thematic analysis. Qualitative Res Sport Exerc Health.

[25] Braun V, Clarke V (2006). Using thematic analysis in psychology. Qualitative Res Psychol.

[26] Bates G, Le Gouais A, Barnfield A, Callway R, Hasan MN, Koksal C, et al. Balancing Autonomy and Collaboration in Large-Scale and Disciplinary Diverse Teams for Successful Qualitative Research. Int J Qual Methods. 2023;22:16094069221144594.

[27] Le Gouais A, Bates G, Callway R, Kwon H, Montel L, Peake-Jones S, et al. Understanding how to create healthier places: A qualitative study exploring the complex system of urban development decision-making [manuscript under review]. 10.1016/j.healthplace.2023.103023 PMC7616384 37079969

[28] Department for Business Energy and Industrial Strategy. Net Zero Strategy: Build Back Greener 2021 [Available from: https://www.gov.uk/government/publications/net-zero-strategy.

[29] Equality Act 2010 c.15. [Internet] Available at: https://www.legislation.gov.uk/ukpga/2010/15/contents. Accessed 6 Feb 2023.

[30] Mayer Brown. NPPF July 2021 Revision, A Guide to What has Changed 27 July 2021 [Available from: https://mayerbrown.co.uk/keep-up-to-date/blog/posts/nppf-july-2021-revision-a-guide-to-what-has-changed/.

[31] Environment Act 2021 c.30. [Internet] Available at: https://www.legislation.gov.uk/ukpga/2021/30/contents/enacted. Accessed 6 Feb 2023.

[32] Healthy Homes Bill [HL] 133 (2022). [Internet] Available at: https://bills.parliament.uk/bills/3139. Accessed 6 Feb 2023.

[33] The Guardian. Charities call for easing of zebra crossing rules to promote walking. 2021.

[34] Public Health and Wellbeing Act 2008, No 46/2008. [Internet] Available at: https://www.legislation.vic.gov.au/in-force/acts/public-health-and-wellbeing-act-2008/053. Accessed 6 Feb 2023.

